# Predictors of positive outcomes from ‘Early Intervention in Psychosis’: protocol for a national retrospective cohort study

**DOI:** 10.3389/fpsyt.2023.1274820

**Published:** 2023-11-03

**Authors:** Ryan Williams, Ed Penington, Veenu Gupta, Apostolos Tsiachristas, Paul French, Belinda Lennox, Alex Bottle, Mike J. Crawford

**Affiliations:** ^1^Department of Brain Sciences, Imperial College London, London, United Kingdom; ^2^Royal College of Psychiatrists, London, United Kingdom; ^3^Department of Psychiatry, University of Oxford, Oxford, United Kingdom; ^4^Department of Psychology, Manchester Metropolitan University, Manchester, United Kingdom; ^5^School of Public Health, Imperial College London, London, United Kingdom

**Keywords:** early intervention, psychosis, schizophrenia, family therapy, CBTp, carer support

## Abstract

**Background:**

Psychotic disorders are severe and prevalent mental health conditions associated with long-term disability, reduced quality of life, and substantial economic costs. Early Intervention in Psychosis (EIP) services aim to provide timely and comprehensive treatment for psychotic disorders, and EIP service input is associated with improved outcomes. However, there is limited understanding of the specific components of EIP care that contribute to these improvements. There is significant nationwide variability in the commissioning and delivery of EIP, with individuals receiving different packages of components from different services. In this study, we seek to explore associations between EIP components and clinically significant outcomes, in order to understand the mechanisms underlying improved psychosis care.

**Methods:**

This national retrospective cohort study will utilize data from the 2019 National Clinical Audit of Psychosis (NCAP), examining the care received by 10,560 individuals treated by EIP services in England. Exposure data from the NCAP, capturing the components of care delivered by EIP services, will be linked with outcome data from routine NHS Digital datasets over a three-year follow-up period. This will be the first study to use this method to examine this population in England. The primary outcomes will be surrogate measures of relapse of psychosis (hospital admission and referral to community-based crisis intervention services). Secondary outcomes include duration of admissions, emergency hospital attendances, episodes of detention under the Mental Health Act, and all-cause mortality. We will use multilevel regression to examine associations between exposures and outcome events. We will handle missing data using appropriate imputation techniques.

**Discussion:**

This study aims to provide valuable insights into the long-term effects of variations in EIP service delivery. The study involves a large, diverse cohort including individuals treated by every EIP service in England. While there are limitations inherent in the observational nature of the study, any associations identified will be of great relevance to clinicians, researchers, and policymakers seeking to optimize EIP care. The results will enable more targeted treatment planning, resource allocation, and potential innovations in EIP care, ultimately leading to improved prognoses for people experiencing psychosis.

## 1. Introduction

Psychotic disorders are highly prevalent, severe mental health conditions that are associated with long-term disability, reduced quality of life and premature mortality (1, 2). They are major contributors to the global burden of disease, and a significant source of expenditure for the United Kingdom economy and National Health Service (3). Current models of care for psychotic disorders stress the importance of intervention early in the course of illness to optimize long-term prognosis (4). Specialized ‘Early Intervention in Psychosis’ (EIP) services were developed to facilitate proactive management of psychotic disorders at an early stage, and have been widely implemented in the UK (5) and internationally (6). These services aim to provide timely and comprehensive treatment, including psychosocial interventions, carer support and medication management with the goal of promoting recovery, reducing hospitalization, and improving outcomes (7).

Despite the widespread adoption and advancement of EIP services, there remains a significant gap in our understanding of the factors within these services that contribute to their observed benefits (8). Individual EIP services differ in the components of care that they deliver, and little is known about how this variation influences outcomes (9, 10). Some components have also been associated with positive results when delivered outside of the typical EIP service framework – for example ‘one-stop network’ services, which are attracting increasing attention as an alternative model of early access mental health service (11).

It is crucial that these associations between specific components of care and favorable outcomes are examined in order to continue to improve the quality of psychosis care. An advanced understanding of these processes would allow for more targeted treatment planning and resource allocation. It may also guide researchers in developing further innovations to enhance the delivery of EIP, and ultimately lead to improved prognoses for individuals experiencing psychosis.

The primary objective of this study is to identify which components of EIP services are associated with improved clinical outcomes for people with psychotic disorders. We will link exposure data from the National Clinical Audit of Psychosis (NCAP) (12) with outcome data from routine NHS Digital datasets, examining the outcomes of 10,560 individuals who were treated by EIP services in England in 2019. There is significant nationwide variability in the commissioning and delivery of EIP, with individuals receiving different packages of components from different services. This project aims to use this variation to examine the effect of specific components of care on outcomes.

## 2. Methods and analysis

This protocol is compliant with the ‘Strengthening the Reporting of Observational Studies in Epidemiology’ (STROBE) statement for observational studies (13).

### 2.1. Study design

This is a national retrospective cohort study. The cohort in question comprises 10,560 individuals for whom data were collected via case-note review as part of the 2019 NCAP (12).

The NCAP is a multi-cycle quality improvement program commissioned by the Health Quality Improvement Partnership (HQIP) on behalf of NHS England. The NCAP has been established as an effective tool to examine the quality of care for people with psychosis. Since 2017 it has been progressively refined over multiple rounds of data collection with input from users and providers of psychiatric services, and provides high quality data on participant demographics (e.g., age, gender, ethnicity, employment/education status) and the components of care that they receive.

In 2019, the NCAP specifically examined all EIP services in England and identified marked variation in components of care at both service and participant levels (12). Individuals received differing packages of treatments (e.g., psychological therapies, carer support). Services also differed in organizational aspects (e.g., waiting times, total caseload, average caseload per care coordinator). The 2019 NCAP received HRA (s215) approval to record patient identifiable data (NHS number/date of birth), enabling linkage with other datasets held by NHS Digital.

We intend to link exposure data from the 2019 NCAP (relating to the components of care delivered by EIP services) with outcome data recorded in routine NHS Digital datasets over the following 3 years. These are the ‘Mental Health Services Data Set’ (MHSDS) recording secondary mental health care provided by NHS Trusts; the ‘Emergency Care Data Set’ (ECDS), and its precursor ‘Hospital Episode Statistics Accident and Emergency’ (HES A&E) recording acute general hospital attendance; the ‘Hospital Episode Statistics Admitted Patient Care’ (HES APC) recording inpatient hospital episodes; and the ‘ONS Civil Registration Death’ recording non-hospital mortality. This linked dataset is currently in production, but not yet available for analysis at the time of publication of this protocol – hence the need for an *a priori* analysis plan.

Using the linked dataset, we will describe the cohort in terms of patient demographics, clinical characteristics, components of care received and outcomes. We will then examine for associations between specific exposures (components of care) and outcomes using appropriate statistical methods. This study has been informed by consultations with service users and carers and their priorities for research.

### 2.2. Exposure variables

Our exposures are specific components of the care provided by EIP services, all of which are specified by NICE as necessary constituents of comprehensive treatment for psychosis (14, 15): receipt of an antipsychotic, receipt of ‘cognitive behavioral therapy for psychosis’ (CBTp), receipt of a family intervention, receipt of vocational support, receipt of a carer focused intervention, offer and initiation of clozapine where appropriate, whether monitoring was conducted with validated outcome measures, receipt of NICE-approved EIP physical health interventions (smoking cessation, weight reduction), EIP service caseload size, care coordinator caseload size, and waiting time (whether waiting time standard was met prior to initiation of treatment).

### 2.3. Outcome variables

Our primary outcome will be time to relapse as indicated by inpatient admission. Secondary outcomes will include time to relapse as indicated by referral to a community-based crisis intervention service, number and length (bed days) of inpatient admissions during the 3-year follow-up period, number of acute general hospital attendances (type 1 emergency departments) during this period, whether any admissions were subject to detention under the Mental Health Act, and all-cause mortality.

### 2.4. Covariates

**Figure 1:**
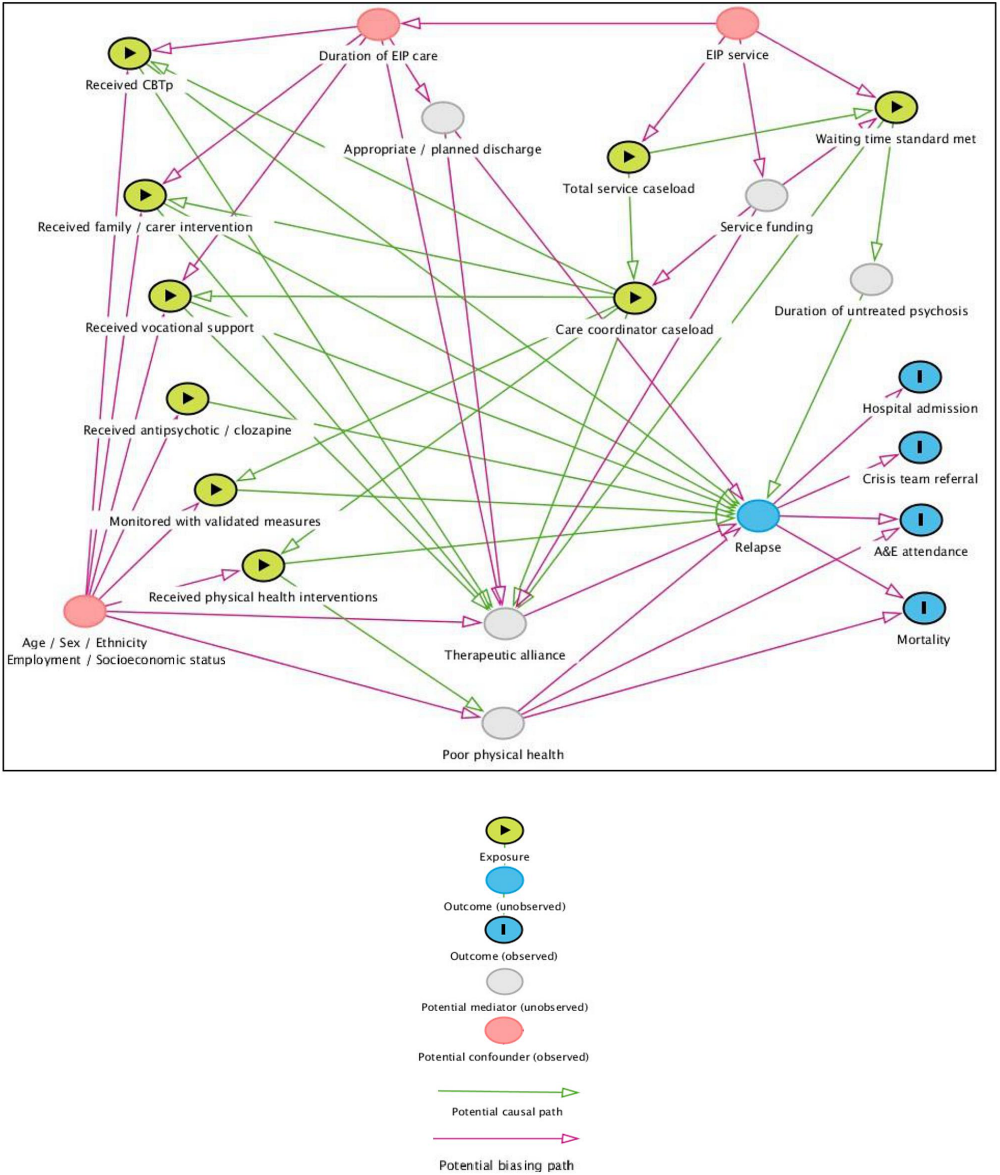
Directed acyclic graph of variables for the proposed analysis.

In preparation for this analysis, we have constructed a Directed Acyclic Graph (DAG) to visually represent hypothesized causal relationships among the variables and covariates in our data (as well as potential unobserved mediators/confounders), in order to guide inclusion in regression models (see Figure 1). This process was informed based on the theoretical expertise of co-authors (including experts in this field and experts by experience) and previous research evidence. Potential confounders which we will be able to adjust for include participant age, sex, ethnicity, employment status and duration of EIP care (at individual level) and EIP service and socioeconomic status of local region (at service level).

### 2.5. Study population

Our sample comprises 10,560 individuals for whom data were collected via case-note review as part of the 2019 NCAP. Data were collected from all 155 EIP teams and from all mental health trusts across all regions in England between June–October 2019. All participating EIP teams provided a complete list of eligible patients to the NCAP audit team, who selected a random sample of 100 patients from each team (where the total caseload included less than 100 eligible patients, all patients were selected).

#### 2.5.1. Inclusion criteria

All participants in the case-note review which was conducted as part of the 2019 National Clinical Audit of Psychosis. Eligibility criteria for the NCAP were as follows:

- Recorded diagnosis of a ‘first episode’ of any ‘non-organic’ psychotic disorder (including affective and substance-induced psychosis).
- Under the care of an EIP service for more than 6 months on 1 April 2019.
- Aged 14–65 – this broad age range reflects current access standards for EIP services recommended by the United Kingdom Royal College of Psychiatrists (16).

#### 2.5.2. Exclusion criteria

Potential participants were excluded from the NCAP if they had a recorded diagnosis of psychosis due to an ‘organic cause’, for example, neurological disorders such as Huntington’s and Parkinson’s disease, dementia, or infections.

### 2.6. Data linkage and storage

We will utilize two sources of data: exposure data from the NCAP and outcome data from NHS Digital (MHSDS, HES, ONS mortality records). Exposure data from the NCAP will be transferred directly to NHS Digital by the Royal College of Psychiatrists (current data controller) and linked to outcome data. The resulting dataset will then be pseudonymized and minimized by NHS Digital to ensure that no patients are identifiable prior to access by our research team. The pseudonymized dataset will be stored within the Office of National Statistics Secure Research Service (ONS SRS).

### 2.7. Statistical analyses

All analyses will be performed using ‘R’ (17). This study involves a comprehensive analysis of the associations between exposure variables and primary and secondary outcomes while accounting for potential confounding factors.

Initially, descriptive statistics will be generated for all exposure variables, outcome measures and covariates as appropriate. Unadjusted tests will then be used initially to explore relationships between the exposure variables, covariates and primary and secondary outcomes.

We will examine associations between exposures and the frequency of outcome events (e.g., number of hospital admissions or acute hospital attendances) using negative binomial regression, in order to account for overdispersion commonly observed in such data. If the frequency of outcome events is small, we will instead dichotomize outcomes and examine associations using logistic regression. We will examine associations between exposures and time to first outcome events (e.g., time to relapse as indicated by admission or referral to crisis support team) using Cox regression. Cox regression allows for the analysis of time-to-event data while accommodating censoring effects, which may occur if participants do not experience the event of interest during the study period.

Multilevel regression models will be used to account for the clustering effects (participants are grouped within EIP services). This approach acknowledges the potential correlation between individuals within the same service, ensuring appropriate adjustments are made to obtain unbiased estimates. All regression models will be adjusted for potential confounding variables as specified in the DAG (participant age, gender, ethnicity, employment/education status and duration of EIP care).

Regarding missing data, our chosen outcomes (e.g., hospitalizations, referrals and use of the Mental Health Act) are considered mandatory submissions for NHS Digital and we anticipate relatively little missing data. Nonetheless, we plan to examine the distribution of any missing data compared with complete data utilizing descriptive statistics and statistical tests, and identify the mechanism of missingness (completely at random, missing at random, not at random).

Following this analysis, we will select an appropriate data handling technique to address missing values from the following options: complete case analysis only; multiple imputation; substituting missing values with mean/modal observed values; using dummy variables as an indicator for missing values. We will perform sensitivity analyses to assess the impact of missing data by comparing the results obtained from different missing data handling techniques. We will transparently report our approach for handling missing data and acknowledge the potential influence of missing data on any interpretations from our findings.

### 2.8. Study power

Considering the dependent variable of ‘hospital admission’, from prior data we estimate that two-thirds of patients treated by EIP teams will have one or more admissions in the 3 years following their referral to EIP (18). As an example, one of our exposure variables would have 5,221 experimental subjects and 5,339 control subjects – data from the NCAP case note audit indicate that 5,221 (49.4%) of patients received CBTp.

For this example, using a 5% level of statistical significance (giving a Type I error probability of 0.05), we estimate that we will have 95% power to detect a small difference in the likelihood of admission to hospital among those who do and do not receive CBTp (equivalent to an odds ratio of 1.05). For reference, the NICE evidence review of CBTp vs. standard care found a RR of 0.76 for rehospitalization up to 18 months following treatment (14).

## 3. Discussion

This study seeks to explore associations between specific components of EIP care and clinically significant outcomes, using a retrospective cohort design. The results of this study will provide valuable insights into the long-term effects of variations in EIP service delivery.

Currently, the literature examining different EIP components is sparse, and there are no comprehensive experimental comparisons of specific components of EIP care. Previous observational comparison studies of EIP services have included relatively few different service models, restricting the opportunity to differentiate components of care (19–21). These studies have also lacked data on real-world outcomes, and been limited by relatively short follow-up times. Although they identified significant variation in outcomes between differing EIP programs, they were ultimately not able to identify any components which accounted for this.

Our cohort is a large, diverse sample encompassing every EIP service in England, and we will have the opportunity to examine a range of clinically relevant, real-world outcome measures over a substantial follow-up period. As such, we would anticipate that results would be widely generalizable with high external validity, and that any associations identified will provide significant information relevant to clinicians, researchers and policymakers seeking to optimize EIP care. To the best of our knowledge, this study is the first to capture and link national audit data with routine outcome data on service use in individuals with mental disorders in England.

This study does have several important limitations. For our exposure variables (i.e., the components of care that were delivered), we are reliant on data provided by the services via the NCAP. However, the NCAP was subject to a vigorous quality assurance process including random data-checking visits to participating trusts by NCAP team members, accompanied by impartial clinicians. Data are therefore of verifiable quality and good reliability. Our primary and secondary outcome measures are surrogate markers of mental wellbeing/relapse (rather than, for example, validated measures of psychotic symptoms). However, they are also objectively important outcomes with clear causal links to mental wellbeing, and clear relevance to patients and clinicians.

As an observational study, this project is also obviously susceptible to inherent limitations such as potential unmeasured confounding and the inability to establish causality. Specific unmeasured confounders include funding variations between services – although we would expect that some of the beneficial effects of improved funding would be mediated by variables that we are examining (waiting times, caseload per care coordinator and availability of interventions). The retrospective design also carries risks of incomplete or missing information. However, we plan to address these limitations through appropriate data handling techniques, and we will transparently report any implications for the conclusions we draw from the results.

In conclusion, this cohort study will provide significant novel data about the processes and outcomes of EIP care. This will help to optimize treatment pathways for people with psychosis and improve quality of life for this vulnerable group.

## Ethics statement

This study was reviewed and approved by y the London Queens Square Research Ethics Committee (REC), part of the NHS Health Research Authority (HRA) – REC reference 22/PR/0602. Written informed consent to participate was not required.

## Author contributions

RW: Conceptualization, Funding acquisition, Methodology, Writing – original draft. EP: Writing – review & editing. VG: Writing – review & editing. AT: Writing – review & editing. PF: Writing – review & editing. BL: Writing – review & editing. AB: Methodology, Supervision, Writing – review & editing. MC: Conceptualization, Methodology, Supervision, Writing – review & editing.
